# Scale-Free Behaviour of Amino Acid Pair Interactions in Folded Proteins

**DOI:** 10.1371/journal.pone.0041322

**Published:** 2012-07-26

**Authors:** Steffen B. Petersen, Maria Teresa Neves-Petersen, Svend B. Henriksen, Rasmus J. Mortensen, Henrik M. Geertz-Hansen

**Affiliations:** 1 Int Iberian Nanotechnol Laboratory INL, Braga, Portugal; 2 Department of Health Science and Technology, Aalborg University, Aalborg, Denmark; 3 The Institute for Lasers, University at Buffalo, The State University of New York at Buffalo, Photonics and Biophotonics, Buffalo, New York, United States of America; 4 Department of Biotechnology, Chemistry and Environmental Engineering, Aalborg University, Aalborg, Denmark; 5 Department of Physics and Nanotechnology, Aalborg University, Aalborg, Denmark; 6 Department of Infectious Disease Immunology, Statens Serum Institute, Copenhagen, Denmark; 7 Technical University of Denmark, Novo Nordisk Foundation Center for Biosustainability Fremtidsvej, Hørsholm, Copenhagen, Denmark; Semmelweis University, Hungary

## Abstract

The protein structure is a cumulative result of interactions between amino acid residues interacting with each other through space and/or chemical bonds. Despite the large number of high resolution protein structures, the “protein structure code” has not been fully identified. Our manuscript presents a novel approach to protein structure analysis in order to identify rules for spatial packing of amino acid pairs in proteins. We have investigated 8706 high resolution non-redundant protein chains and quantified amino acid pair interactions in terms of solvent accessibility, spatial and sequence distance, secondary structure, and sequence length. The number of pairs found in a particular environment is stored in a cell in an 8 dimensional data tensor. When plotting the cell population against the number of cells that have the same population size, a scale free organization is found. When analyzing which amino acid paired residues contributed to the cells with a population above 50, pairs of Ala, Ile, Leu and Val dominate the results. This result is statistically highly significant. We postulate that such pairs form “structural stability points” in the protein structure. Our data shows that they are in buried α-helices or β-strands, in a spatial distance of 3.8–4.3Å and in a sequence distance >4 residues. We speculate that the scale free organization of the amino acid pair interactions in the 8D protein structure combined with the clear dominance of pairs of Ala, Ile, Leu and Val is important for understanding the very nature of the protein structure formation. Our observations suggest that protein structures should be considered as having a higher dimensional organization.

## Introduction

A key challenge for protein science is to understand the structure and dynamics of the complex web of interactions in proteins that contribute to the 3D structure and function. Proteins attain their function through their 3D structure which is the cumulative result of numerous interactions between amino acid residues interacting with each other through space and/or chemical bonds. 1288 different folds have been identified [1], [2]. Kauzmann [3], Bernal [4] and Tanford [5], [6] proposed that the hydrophobic effect drives protein folding. The speed at which the protein attains its folded state is staggering: out of a near infinitude of possible ways to fold, a protein picks one in just tens of microseconds. Levinthal [7] speculated in 1969 that if a 100 amino acid protein has 3 conformational states available for each of the two dihedral angles in each of the 99 peptide linkages then the protein needs to explore 3^198^ conformational states if it searches the conformational space exhaustively. If each state can be explored in 1 picosecond (a characteristic time for a bond vibration), then the protein needs more time than the age of the universe in order to search all conformational states exhaustively. Yet we know that a protein attains its structure in a matter of milliseconds. Levinthal concludes that a guiding mechanism or principle must be available to the protein. Four decades ago, C.B. Anfinsen hypothesized that “information dictating the native fold of protein domains is encoded in their amino acid sequence” [8]. Despite the explosive growth in the number of high-resolution 3D protein structures, the elusive “fold code” has not been identified [9]–[14]. Several models exist for the folding mechanism of proteins. In the nucleation-condensation model the folding is initiated by the formation of a meta-stable transition state [15]. The transition state, called the nucleus, consists of a particular pattern of amino acid contacts and serves as template for the rapid structure condensation. The concurrent buildup of secondary and tertiary contacts is a defining feature of this model which contradicts the hydrophobic collapse model. Here proteins fold via an initial collapse driven by hydrophobic effects. Secondary structural elements are formed in the collapsed state early in this process, condensing into the tightly packed tertiary structure. Protein denaturation induced by organic solvents is consistent with hydrophobicity being essential for the fold. Both models point to the fact that the protein structure has both a local and non-local sequence component, and that a protein’s secondary structure is as much a consequence of the tertiary structure as it is a cause of it [12], [13]. Protein structure is not likely to be dominated by electrostatic interactions among charged residues because most proteins have relatively few charged residues and they are concentrated in high-dielectric regions on the protein surface.

A possible approach to analyzing a protein structure is to perceive it as the cumulative result of all interactions between amino acid pairs in the protein. Higher order contributions involving more than 2 amino acid residues are possible as well, but in this first approach we will limit ourselves to pair interactions. Several parameters influence the contribution of the amino acid pair interaction to protein stability. In the present work we quantify the interaction between two amino acid residues in terms of 8 parameters: the type of each amino acid residue interacting (AA1, AA2), their solvent accessibility, the secondary structural element where they are located (SS1, SS2), the protein size (sequence length), the sequence and spatial distances between the interacting amino acid residues. We have established an 8-dimensional tensor compiling information about the 3D structural environment of all pairs of amino acids found in 8706 protein structures in terms of the 8 parameters described above. This hyper dimensional tensor represents our model of protein structural space. Each cell in the resulting 8D tensor contains the number of times a pair of two particular amino acids has been found in a location in the higher dimensional space. A highly populated hyper cube cell indicates that that particular type of amino acid pair is found repeatedly in exactly the same location in the 8 dimensional space. Of some reason Nature has found this particular constellation optimal for protein structure formation.

In the present paper we present a detailed analysis of the 8D data cube. We show that the amino acid pair interactions in folded proteins are consistent with a scale free organization model. In recent years there has been a rapidly growing understanding of the rules that guide self-organization of various structures such as the World Wide Web [16], metabolic networks as well as disease networks [17], [18]. In all these cases a scale free organization that seemingly forms spontaneously is reported. It appears prudent to see the scale free organization as a guiding principle for self-organizing structures. We here report that exactly the same phenomenon is found for protein structures. We suggest that achieving a particular protein structure and securing protein stability should be considered as a process taking place in a higher dimensional space and that pairs formed among the amino acids Ala, Ile, Leu and Val form highly populated structural nodes in the final structure. We believe our findings provide new insight into key interactions essential to protein structure and structural stability.

## Results

### Scale Free Behavior

**Figure 1:**
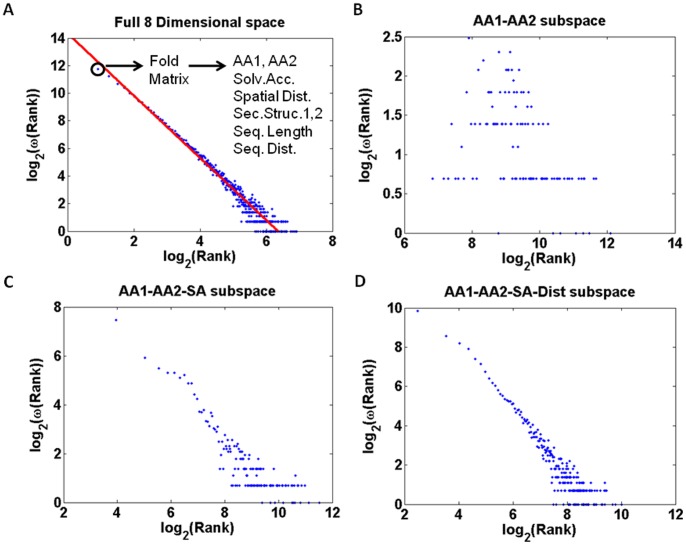
The fold matrix used in the present study is 8 dimensional. Its content can be projected onto any subspace one may define. The cell content (rank or number of amino acid pairs) is plotted against the frequency that such a rank is found in a log-log plot: in (A) is shown the log-log plot for the full 8 dimensional fold matrix. A linear fit to the points resulted in a slope of −2.26±0.05 and an intercept at 14.3, (B) depicts the 2-dimensional amino acid type subspace data, (C) the 3 dimensional subspace consisting of 2 amino acid types and 1 solvent accessibility dimension, and (D) the 4 dimensional subspace consisting of 2 amino acid types, 1 solvent accessibility and 1 distance dimension.

The topology of the protein high dimensional space is characterized by densely populated clusters as well as sparsely populated regions. While the maximum population of a cell is 1004, the average cell population is 3.12 counts. We have investigated the nature of this spread by analyzing the values found in the non-zero cells of the matrix. When plotting cell population against the number of cells that has the same population size, a scale free behavior is found: ω(R) = R^−λ^, where R is the rank or population of a cell, and ω(R) is the number of times such a cell population was encountered [23]. When plotting log_2_(ω(R)) against log_2_(R), a straight line with slope −2.333 is obtained [Fig. 1(A)]. Any given point in the scale free plot corresponds to a set of cells in the matrix that share the same population (rank). Given that we know the set, we can extract which location(s) in the 8 dimensional space that contributed to the set, in terms of amino acid types, solvent accessibility, Euclidian distance, secondary structures as well as sequence distance and protein length.

In principle the scale free organization could originate from the distribution of amino acid pairs. Collapsing the 8D matrix information onto the 2D amino acid plane (only AA1 and AA2 dimensions are included) resulted in 400 cells that differed strongly from scale free behavior [Fig. 1(B)]. When expanding the subspace to include the solvent accessibility dimension we observe some indications of scale free behavior [Fig. 1(C)]. Adding the Euclidian distance dimension it seems possible that the 4 dimensional data is organized in a scale free manner [Fig. 1(D)]. Including all 8 dimensions, the scale free feature becomes very distinct and well defined [Fig. 1(A)]. In order to verify such claims we have fitted the data. When using the 3 dimensions AA1, AA2, and SA [Fig. 1(C)] and fitting the data, a straight line with slope −1.104 is obtained (RMS of 0.893). When using the 4 dimensions AA1, AA2, SA and Dist [Fig. 1(D)] and fitting the data, a straight line with slope −1.650 is obtained (RMS of 0.952). The value of the exponent lambda is now closer to the expected value for a scale free network, where λ is generally between 2.1 and 3 [23]. The RMS of the fit has definitely improved upon adding Dist as the 4^th^ dimension. When using the 8 dimensions [Fig. 1(A)] and fitting the data, a straight line with slope −2.333 is obtained (RMS of 0.988).

Working with the full 8 dimensional dataset allows us to extract all protein structural features for a given cell. Thus, for any particular rank, we are able to identify amino acid types, solvent accessibility, Euclidian distance, secondary structures as well as sequence distance and protein length.

We have analyzed which minimal subset of observables in our 8-dimensional space would still give a reasonable scale-free approximation with an exponent lambda closest to the value of 2.3 extracted from the full data set. Here we provide a sorted list of the fit results that resulted in a fit with better or equal than 0.980 RMS deviation. Data is displayed in Table 1. The left text column indicates which dimensions were included: AA1, AA2 (amino acid1 and 2); SA (solvent accessibility); Dist (Cartesian distance); SS1 and SS2 (secondary structure for amino acid 1 and 2); Plen (protein length) and Seq Dist (sequence distance). The following column is the slope = −(exponent lambda). Finally the RMS value for the fit is given in the extreme right column. As judged by the RMS value the best fit is obtained for the 7 dimensional case: AA1 AA2 SA Dist SS1 PLen SeqDist. However, the improvement is marginal compared to the 8 dimensional case.

**Table 1:** Dimensions that lead to a fit with better or equal than 0.980 rms deviation.

Dimensions	Slope = −exponent λ	RMS
AA1 AA2 SA Dist SS1 PLen SeqDist	−2.291 (−2.312 −2.270)	0.992
AA1 AA2 SA Dist SS2 PLen SeqDist	−2.291 (−2.312 −2.270)	0.992
AA1 AA2 SA SS1 SS2 PLen SeqDist	−2.279 (−2.301 −2.256)	0.991
AA1 AA2 Dist SS1 SS2 PLen SeqDist	−2.279 (−2.301 −2.256)	0.991
AA1 AA2 SA Dist SS1 PLen	−2.220 (−2.242 −2.197)	0.990
AA1 AA2 SA Dist SS2 PLen	−2.220 (−2.242 −2.197)	0.990
AA1 AA2 SA Dist SS1 SS2 PLen SeqDist	−2.333 (−2.360 −2.307)	0.988
AA1 AA2 SA Dist SS1 SS2 PLen	−2.296 (−2.322 −2.270)	0.988
AA1 AA2 SA SS2 PLen SeqDist	−2.186 (−2.211 −2.160)	0.987
AA1 AA2 Dist SS1 PLen SeqDist	−2.186 (−2.211 −2.160)	0.987
AA1 AA2 SA SS1 PLen SeqDist	−2.165 (−2.191 −2.139)	0.987
AA1 AA2 Dist SS2 PLen SeqDist	−2.165 (−2.191 −2.139)	0.987
AA1 AA2 SA Dist SS1 SeqDist	−2.148 (−2.177 −2.119)	0.983
AA1 AA2 SA Dist SS2 SeqDist	−2.148 (−2.177 −2.119)	0.983
AA1 AA2 SA SS1 SS2 PLen	−2.189 (−2.219 −2.159)	0.982
AA1 AA2 Dist SS1 SS2 PLen	−2.189 (−2.219 −2.159)	0.982
AA1 AA2 Dist PLen SeqDist	−2.008 (−2.038 −1.978)	0.981
AA1 AA2 SA PLen SeqDist	−2.009 (−2.039 −1.979)	0.981
AA1 AA2 SA SS1 SS2 SeqDist	−2.073 (−2.103 −2.043)	0.980
AA1 AA2 Dist SS1 SS2 SeqDist	−2.073 (−2.103 −2.043)	0.980

The left text column indicates which dimensions were included: AA1, AA2 (amino acid1 and 2); SA (solvent accessibility); Dist (Cartesian distance); SS1 and SS2 (secondary structure for amino acid 1 and 2); Plen (protein length) and Seq Dist (sequence distance). The following column is the slope = −(exponent λ). Lambda ranges from 2.008 to 2.333. Following this column is the 95% confidence interval for the exponent in parenthesis. Finally the RMS value for the fit is given in the extreme right column.

**Figure 2:**
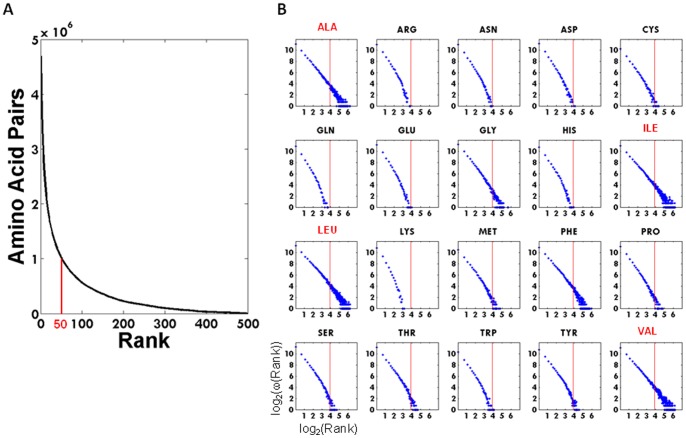
Enumeration of amino acid pairs. (A) Cumulative number of amino acid pairs found above a given rank. (B) Log log plots of the individual amino acid as a function of rank 1∶300. The vertical line is rank 50. The maximum rank achieved by the individual residues were Alanine (298), Arginine(46), Asparagine(49), Aspartate(61), Cysteine(78), Glutamine(40), Glutamate(55), Glycine(295), Histidine(53), Isoleucine(298), Leucine(299), Lysine(24), Methionine(122), Phenylalanine(294), Proline(130), Serine(113), Threonine(110), Tryptophan(60), Tyrosine(99), Valine(300).

In Figure 2(A) is displayed the cumulative number of amino acid pairs found above a given rank. In Figure 2(B) we investigate if the individual pairs containing a particular amino acid behave similarly to the cumulative behavior depicted in Figure 1(A), by displaying the log log plots of the individual amino acid as a function of rank (from 1∶300). All 20 curves can be interpreted as having a scale free nature. The vertical red line corresponds to rank 50. Alanine, Isoleucine, Leucine and Valine display a linear behavior in the log-log plot, whereas the titratable residues Arginine, Lysine, Aspartate, Glutamate, Histidine and Cysteine exhibit a power law with exponential cutoff. After making a linear fit to each curve displayed in Figure 2(B), the intersection with the log_2_(Rank) axis could be determined. The maximum rank (cell population) achieved by the individual residues were Alanine (298), Arginine(46), Asparagine(49), Aspartate(61), Cysteine(78), Glutamine(40), Glutamate(55), Glycine(295), Histidine(53), Isoleucine(298), Leucine(299), Lysine(24), Methionine(122), Phenylalanine(294), Proline(130), Serine(113), Threonine(110), Tryptophan(60), Tyrosine(99), Valine(300).

### Amino Acid Pair Preferences

**Figure 3:**
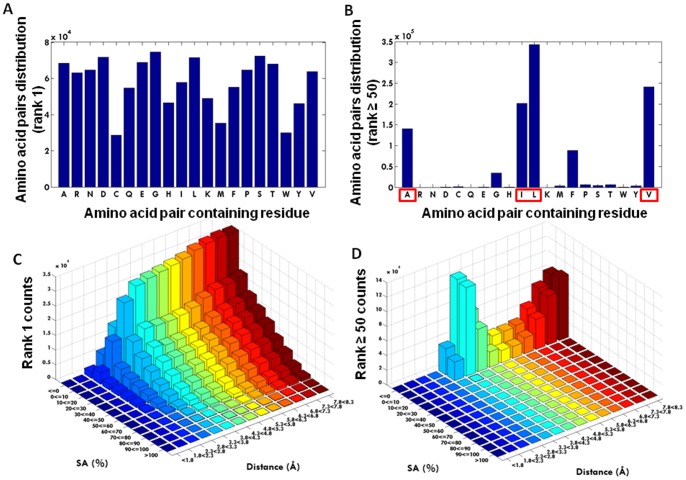
Amino acid pair distribution. (A) Distribution of the amino acid pair containing residues for rank 1 cells (1.15*10^6^ amino acid pairs); (B) Distribution of the amino acid pair containing residues for rank ≥50 cells (1.07*10^6^ amino acid pairs). Each amino acid is represented by its one letter code; (C) 2D histograms of Euclidian distance between the amino acids in a pair *vs* solvent accessibility seen for rank 1 cells; (D) 2D histograms of Euclidian distance between the amino acids in a pair *vs* solvent accessibility seen for rank ≥50 cells.

**Figure 4:**
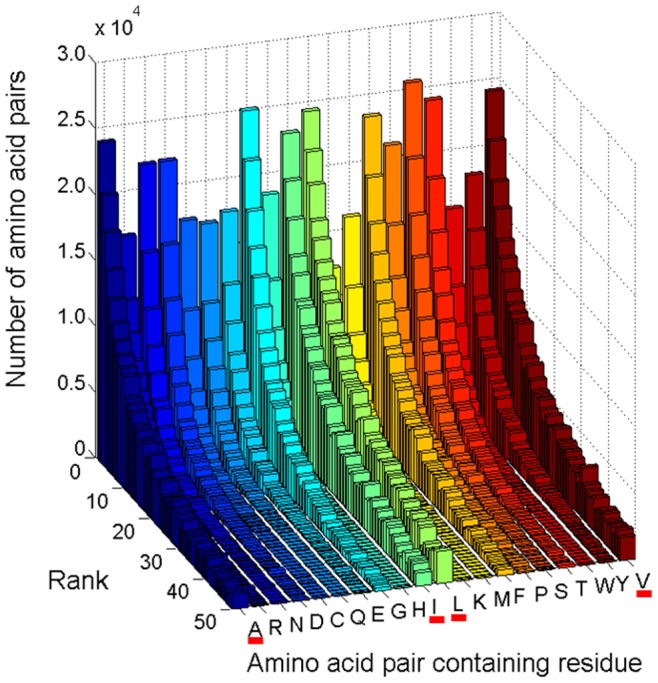
Number of amino acid pairs containing each specific amino acid residue as a function of cell rank. The residues that have a large number of links to other residues are underlined in red (Ala, Ile, Leu and Val).

In Figure 3A is displayed the amino acid pairs distribution for rank 1 (1.15*10^6^ amino acid pairs) while in Figure 3B is displayed the amino acid pairs distribution for rank ≥50 (1.07*10^6^ amino acid pairs). Each amino acid is represented by its one letter code. 2D histograms of Euclidian distance (in Å) between the amino acids in a pair *vs* solvent accessibility (SA) seen for rank 1 and for rank ≥50 are displayed in Figure 3(C) and 3(D), respectively. Data shows that cells of rank 1 display no single amino acid preference [Fig. 3(A)]. In contrast, when analyzing which amino acid pairs contributed to the cells with a population above 50 (rank 50), 4 of the 20 amino acids dominate: Alanine, Isoleucine, Leucine and Valine [Fig. 3(B)]. Such pairs are preferentially buried in protein structures (SA ≤10) in a spatial distance of 3.8–4.3Å [Fig. 3(D)] and in a sequence distance >4 residues (data not shown). In contrast to what was observed at rank ≥50, no focal preferences are observed in the rank 1, SA *versus* Distance subspace [Fig. 3(C)]. It is clear from the graph that the buried state is preferred, and that the majority of pairs are observed with a Euclidian distance exceeding 3.8Å. In Figure 4 is displayed the number of amino acid pairs containing each specific amino acid residue as a function of cell rank. It can be observed that only the curves containing Alanine, Isoleucine, Leucine and Valine remain populated at high rank values. It is crucial for a proper understanding of the 8D matrix, that one can search for a particular cell or cell content as is the case for the 1004 Leu Leu pairs, or one can project the full 8D matrix onto a subspace, such as a subspace defined by the solvent accessibility and the metric distance between the two amino acids (see e.g. figure 3C and D). Whereas Figure 3B have taught us that amino acid pairs containing Ala, Ile, Leu and Val dominate the rank ≥50 set (and contains more than 1 million amino acid pairs), followed by Phe and Gly, Figure 3D is a projection of the 8D data matrix onto the subspace SA – distance. More than 1 million amino acid pairs are contacts made between the four mentioned residues thus constituting more than 18% of the total amount of amino acid pairs.

**Figure 5:**
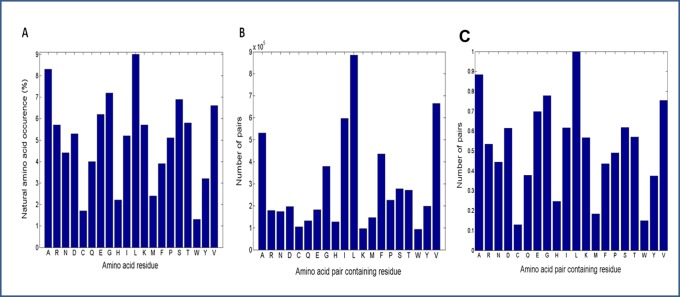
Natural occurrence of amino acid residues in proteins (A), occurrence of amino acid pairs containing a particular amino acid residue retrieved from the 8D matrix (B) and occurrence of amino acid pairs containing a particular amino acid residue retrieved from the randomized reference 8D matrix (C).

In Figure 5 is displayed the natural occurrence of amino acid residues in proteins and the occurrence of amino acid pairs containing a particular amino acid residue retrieved from the 8D matrix. It is observed that despite the higher occurrence of the four residues (Ala, Ile, Leu and Val) in amino acid pair interactions in proteins [Fig. 5(B)], they are not the four most abundant residues in proteins [Fig. 5(A)]. The four most abundant amino acid residues are Leu, Ala, Glu and Pro, followed by Val and Gln. The normalized abundance plot of the randomized dataset is displayed in Fig. 5C. It can be seen that upon randomization the distribution plot reflects the natural occurrence of the amino acids in proteins [Fig. (5A)]. The clear preference for Ala, Ile, Leu and Val in amino acid pairs is not observed in Figure 5C while it was clear in Figure 5B. In Figure 5C the residues that display a larger number of pairwise interactions are Leu, Ala, Gly, Val, Glu and Asp(Ile or Ser).

**Figure 6:**
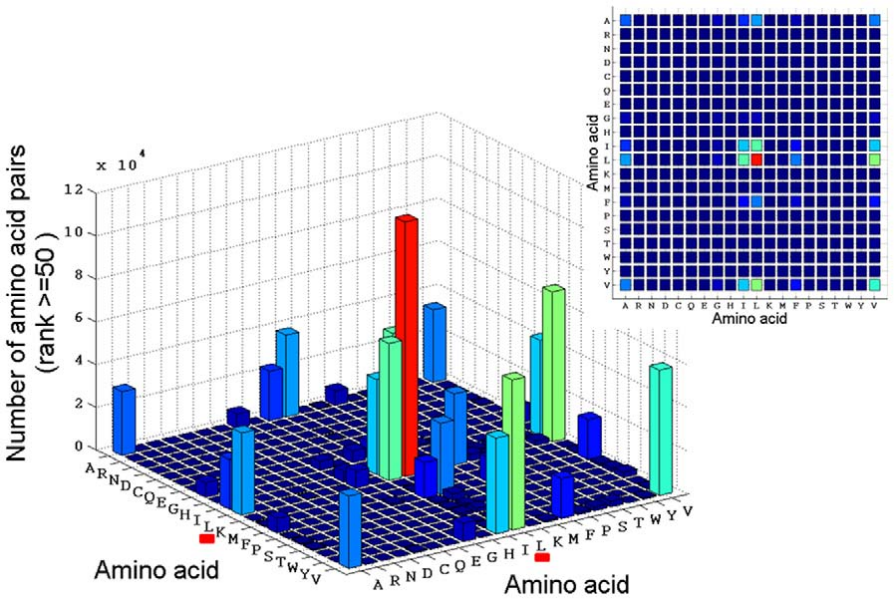
Number of amino acid pairs and the type of amino acid forming pairs in cells with population ≥50 (rank 50). As an insert, is displayed the 2D projection of the same graph.

**Figure 7:**
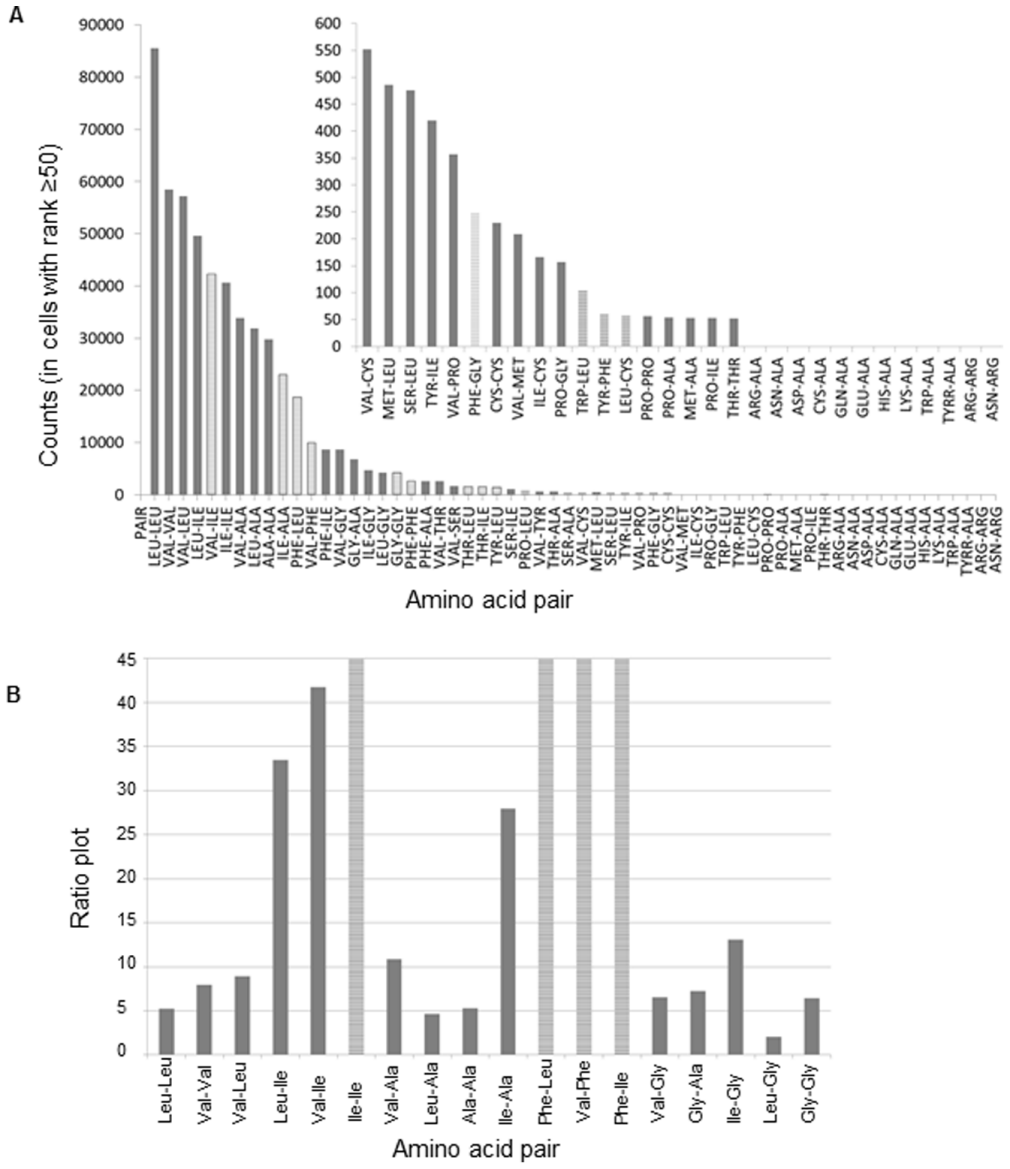
Pairwise preferred interactions. (A) Number of pairs in the 8D matrix (non-randomized, cells with rank ≥50). The insert shows in detail the distribution of the pairs not clearly seen in the main panel (from pair VAL-CYS onwards). (B) Ratio plot between the “number of pairs in the 8D matrix” and the “number of pairs in the randomized 8D matrix” (for the cells with rank ≥50).

**Figure 8:**
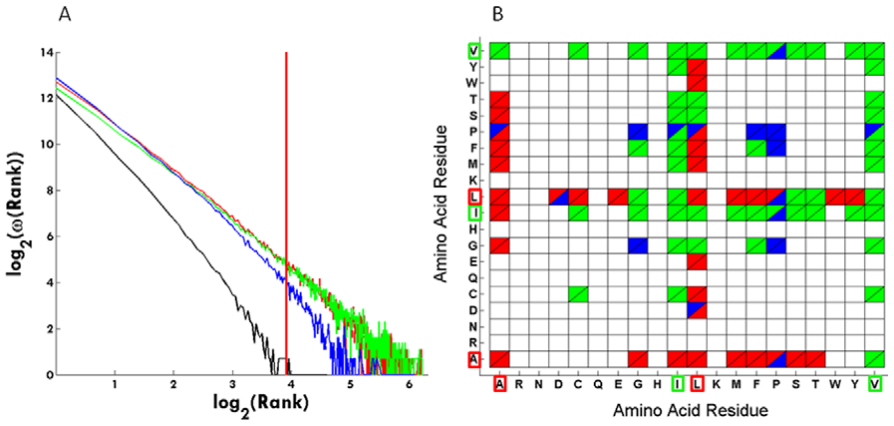
Secondary structural preferences. (A) The graph shows how the secondary structure assignment changes with rank. Red codes for alpha-helix, green for beta-strand, blue for coil and black for turn. The vertical red line is located at rank 50. (B) Amino acid pairs and their secondary structural location in cells with population ≥50 (rank 50). Color scheme is the same as described for panel (A). Ala, Ile, Leu and Val residues are highlighted with a red or green box.

The resulting 8 dimensional matrix contains 77.41 million cells. In our analysis of 8670 protein 3D chains, 5.89 million amino acid pair observations were found and loaded into 1.89 million cells in the matrix. Each of these cells contains the number of times a pair of two particular amino acids has been found in a location in the 8D space. In many cells only a single observation has been allocated, whereas the Leu-Leu pair scores the highest single cell count: 1004. In Figure 6 is displayed the number of amino acid pairs and the type of amino acid forming pairs in cells with population ≥50 (rank 50). It can be observed that the Leu-Leu pair is the most abundant pair [Fig. 6, red column and Fig. 7(A)]. We can deduce from the cell coordinates that this pair is found in a buried location with a Euclidian distance of 3.8–4.3Å. Both residues are located in α-helices [see Fig. 8(B)], in an average length protein (300–400 AA) and in a sequence distance exceeding 4 residues (data not shown). An insert is displayed with a 2D projection of the same graph. It can be observed that pairs among Ala, Ile, Leu and Val are dominant in cells with population ≥50 (rank ≥50). Interestingly, all four residues display a larger number of contacts with Leu. Figure 7(A) shows the number of pairs observed in cells with rank ≥50. Clearly it can be seen that the 10 most abundant pairs involve the residues Leu, Val, Ile and Ala. The next three most abundant pairs involve interactions between Phe and the three most hydrophobic residues: Phe-Leu, Phe-Val and Phe-Ile.

In order to determine the statistical significance of the coordinates of the observed pairs, the ratio between the actual findings and the randomized value has been plotted [see Fig. 7(B)]. It can be observed that the found pairs are statistically significant since they are more frequent in the actual protein database than in the randomized reference database. Interestingly, the most abundant pairs are Ile-Ile, Val-Ile, Leu-Ile, Ile-Ala and to Phe-Leu, Phe-Val and Phe-Ile followed by pairs between Gly and Val, Ala and Leu. The bars with stripes displayed in Figure 7B are highly significant since they are abundant in the protein 8D matrix but have not been seen in the randomized reference dataset, which leads to an infinite ratio. For example, 40638 Ile-Ile pairs, 9915 Phe-Val pairs, 8724 Phe-Ile pairs, and 8724 Ile-Phe pairs are seen in the 8D matrix (in those cells with more than 50 amino acid pairs, i.e. rank ≥50) while none of these pairs are seen in the randomized reference 8D matrix.

### Secondary Structural Preferences

Figure 8A shows how the secondary structure assignment changes with rank. The log_2_ of the cell population against the log_2_ of rank is displayed. Red codes for alpha-helix, green for beta-strand, blue for coil and black for turn. A scale free organization is observed also at the secondary structural level. The alpha and beta categories appear almost linear in the log-log plot, whereas both the coil and turn categories appear to follow a power law with exponential cutoff. At rank = 1 all 4 types of secondary structures are well populated. When the rank increases, all secondary structures become less populated, but the turn and coil categories lose population faster than the alpha and beta categories. The vertical red line is located at rank 50. When analyzing which amino acid pairs contributed to the cells with a population above 50 (rank ≥50), we observe that the above mentioned preferred pairs of Alanine, Isoleucine, Leucine and Valine are preferentially located in either alpha-helices (red curve) or beta-strands (green curve). They are also seen in coil elements (blue curve) but not in turns (black curve). In Figure 8B are displayed the secondary structure preferences of each amino when in a pair located in a cell with a population equal or above 50 (rank ≥50). Data shows that the pairs containing the highly connected residues (Alanine, Leucine, Isoleucine, Valine) have clear secondary structural preferences. For example, pairs containing Alanine and Leucine are predominately located in alpha-helices whereas pairs containing Isoleucine and Valine are preferentially located in beta-strands. Further details are displayed in Figure 8B.

**Figure 9:**
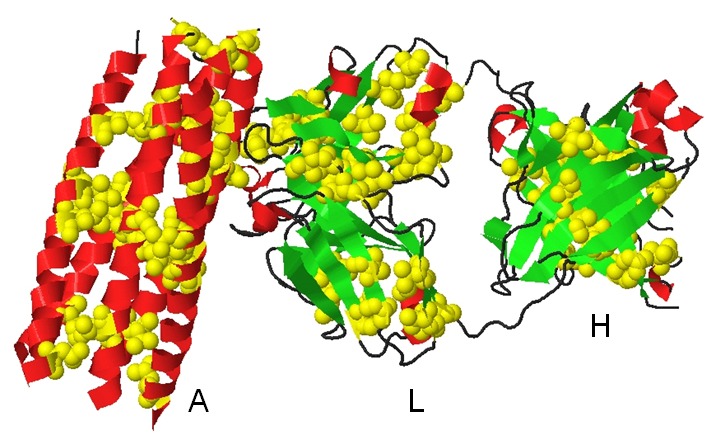
Visualizing amino acid pairs containing Ala, Ile, Leu and Val residues. Crystal structure of the N-heptad repeat of HIV-1 gp41 mimetic 5-helix complexed with two antibody fragments (3MA9.pdb). Amino acid pairs containing Ala, Ile, Leu or Val residues are highlighted in yellow and as CPK. Alpha-helices are colored red and beta-sheets green. The three different chains are displayed: A (HIV-1 gp41 5-helix), L and H (Fab fragments).

In Figure 9 are highlighted the amino acid pairs containing Ala, Ile, Leu and Val residues in the 3MA9.pdb structure, which is the crystal structure of the N-heptad repeat of HIV-1 gp41 mimetic 5-Helix complexed with two antibody fragments (Fab). The distance between the displayed paired residues was limited to 3.8–4.8Å [please see Fig. 3(D)]. Residues with solvent accessibility ≤20% were displayed in yellow. It can be seen that the pairs containing Ala, Ile, Leu or Val residues connect different secondary structural elements (alpha-helices and beta-sheets). Pairs containing these residues located at the end of beta-strands link those strands (chain L). The displayed pairs are the ones with special the characteristics displayed in Figure 3D.

## Discussion

Protein structure emerges from the sum of the interactions among the different amino acid residues. These interactions are responsible for protein function. A major challenge of protein science is to embark on an integrated theoretical and experimental program to map out, understand and model in quantifiable terms the topological and dynamic properties of the inner protein structure network that is responsible for protein structural stability and protein function. Rapidly developing theory of complex networks (e.g. social, technological) is proving quite useful in understanding e.g. biological networks, including protein-protein interactions, metabolic, signaling and transcription-regulatory networks. Like the structure of a protein is a result of the sum of the interactions among the different amino acid residues, various types of networks, or interaction webs such as protein-protein interactions and other cellular networks, emerge from the sum of the interactions among different molecules.

It is necessary for the discussion of the results presented in this paper that the concepts of random networks, scale-free networks, degree of a node, power-law, and hub are introduced. We will start by describing briefly 2 out of 3 types of network models, which are crucial for understanding complex networks and help to explain the origin of observed network characteristics. All 3 models have a direct impact on our understanding of biological networks [24]. Random networks are characterized by the fact that most nodes have approximately the same number of links and the node degrees follow a Poisson distribution. Degree of a node in a network is the number of connections it has to other nodes. The tail (high k region) of the degree distribution P(k) decreases exponentially, which indicates that nodes that significantly deviate from the average are extremely rare. One of the earliest observations relevant to the topology of a large e.g. protein-protein interaction network was that it possesses the “scale-free” property, i.e., the nodal degree distribution of the network is a power-law distribution [23]–[25]. Power-law is a function f(x) where the value y is proportional to some power of the input x, y = f(x) ∼x^−α^. Degree distribution is the probability distribution of the degrees over the whole network. In a scale-free network the probability that a node has k links follows P(k) α k^−γ^. Hub is the name given to a few nodes which establish a large number of links.

When analyzing direct physical interactions in protein-protein interactions networks for example, it is observed that most proteins interact with only a few other proteins while a small number of proteins (hubs) have many interaction partners [26]. So, it is the high frequency of interactions by a small numbers of proteins the reason for calling that group of small proteins the “hubs”. It is also reported that the per protein distribution of the interactions follows an inverse power law [27], indicating a typical scale-free network topology [19]–[24]. Most cellular networks within the cell approximate a scale-free topology. The first evidence came from the analyses of metabolism, in which the “nodes” are the metabolites and the “links” the enzyme-catalysed biochemical reactions. The analyses of the metabolic networks of 43 different organisms from all three domains of life (eukaryotes, bacteria, and archae) indicate that the cellular metabolism has a scale-free topology, in which most metabolic substrates participate in only one or two reactions, but a few, such as pyruvate and coenzyme A, participate in dozens and function as metabolic hubs [17], [18]. In our study, we consider that each amino acid residue is a “node” and we observe that 4 amino acids establish many more pairwise interactions with other residues than the other 16 amino acid residues. We define a through space interaction between 2 amino acid residues as the “link”. The probability that a node (amino acid) has *k* links follows a power-law distribution P(k) α k^−γ^. Therefore, the amino acid network in a protein is a scale-free network. In our manuscript we reported that when plotting the cell population observed in the 8D matrix (number of amino acid pairs observed in each cell) against the number of cells that has that same population size, we observe a scale-free distribution [Fig. 1(A)]. This means exactly the same as in the previous text, just with other words. Furthermore, a new interesting new observation if that this scale free distribution is only observed when several dimensions are included (Fig. 1 and Table 1). The 8 dimensions are: the type of each amino acid residue interacting (AA1, AA2), their solvent accessibility, the secondary structural element where they are located (SS1, SS2), the protein size, the sequence and spatial distances between the amino acid residues interacting. So, a protein network is observed to be a scale-free network only if we take into account the many dimensions of the network. The four residues or “nodes” that have the highest number of “links” are 4 amino acid residues: Ala, Ile, Leu and Val [see Fig. 3(A,B)]. The highest-degree nodes in a network are usually called “hubs”, and they are thought to serve specific purposes in a network. It has been a common practice in the analyses of protein interaction networks to define an *ad hoc* threshold or degree scale such that all nodes (proteins) that have degree higher than this threshold are considered to be special in some sense and are called “hub” nodes. We could attempt to call these 4 amino acid residues “hubs”. However, since no threshold has yet been defined, we prefer to simply state that these 4 “nodes” have a high degree since they establish a large number of connections to other nodes.

The present study has unraveled the preferential occurrence of amino acid pairs in protein structures in a local context in a particular solvent shell. Clearly the organization of such pairs is very specific if it involves pairs of Ala, Leu, Ile or Val [Fig. 3(B,D)], and very diffuse and unspecific if the pair is composed of the other residues [Fig. 3(A,C)]. Based on these observations it appears plausible that well defined pairwise interactions essential to protein structure and protein stability are formed by the 4 residues Ala, Leu, Ile and Val, followed by Phe and Gly [Fig. 3(B) and Fig. 7(A)]. Our observations may explain why predicting protein 3D structures from amino acid sequence alone, has proven so hard. Any rule is based on statistical evidence – but since our results indicate that amino acid pair interactions are organized in a scale free manner, only a few amino acid pairs occur sufficiently frequent, such that a rule can be constituted. Extracting high incidence occurrences, such as the Leu-Leu pairs mentioned above (Fig. 6), would represent observations that could be transformed into a ’rule’. If we want more than 500 occurrences, before we regard the observation as statistically sound, we will only cover around 26000 pairs in the matrix. If we reduce stringency to 50 occurrences, we will cover about 1.02 million pairs, or less than 20% of the total [Fig. 2(A)].

The observed large connectivity among the 4 residues (Ala, Ile, Leu, Val) observed in our present work is supported by the findings of Buchete et al. [28]. They present a quantitative analysis of the amino acids pair distributions and their associated contact potentials, comparing some of the contact potentials developed to date. Furthermore, they rank quantitatively the importance of various inter-residue interactions. Pairwise contact potentials are widely used representations of inter-residue interactions and have been successfully used in many applications ranging from protein structure prediction to protein design and docking. Buchete et al. [28] observe that most contacts occur between the small-hydrophobic amino acid residues (Ala, Ile, Leu, Val, Met). Both works point at that Ala, Ile, Leu, Val are part of a “reduced folding alphabet” of some of the most used contact potentials such as those of Miyazawa and Jernigan [29]. Our analysis done here has several novel points - most notably the clear observation of the scale-free relationship behind the distribution of pairwise interactions. This has never been reported before. The work by Miyazawa and Jernigan [30] estimates the effective inter-residue contact energies for protein in solution from the numbers of residue-residue contacts observed in crystal structures of globular proteins. They report that the residues with the largest amount of surrounding residues are (Gly, Val, Ala, Leu, Ser, Ile). In our studies, we report that the residues that establish the largest number of pairwise interactions are (Leu, Val, Ile, Ala, followed by Phe and Gly). Both works point at that Ala, Ile, Leu, Val are among the residues that establish a large number of inter-residue contacts in proteins, despite the fact that these are not the 4 most abundant residues in proteins (see Figure 5, panel A – the most abundant residues in proteins are Leu, Ala, Glu, Pro, Val, Gln). Our work not only shows which residues establish the largest number of pairwise interactions but also we report what are the residues that they pair with. Furthermore, we show that there is a scale-free relationship behind the distribution of pairwise interactions. When comparing our finding with the results from Miyazawa and Jernigan [30], we do see that both works report that the residues that establish the largest number of contacts among themselves are (Leu, Val, Ile, Ala, Phe, Gly). This can be seen in our present work in Figure 7A and in the work published by Miyazawa and Jernigan [30], Table IV therein].

In the present work the scale free organization of the frequency of the number of interactions (pairs) established by each amino acid residue is shown (Fig. 1). Data has pinpointed the importance of the four amino acid residues that establish the larger number of interaction [see Fig. 3(A,B)]. Pairs of Ala, Leu, Ile, Val and their mixed pair combinations thereof form the highly connected nodes of the protein and occur many times, whereas all other amino acid pairs occur fewer times (see Fig. 7). It is important to stress that the scale free organization of the amino acid pair interactions is only observed in a higher dimensional space (Fig 1 and Table 1), and it may differ topologically from many of the other lower dimensional networks addressed in literature [31]. Figure 1B shows that when only AA1 and AA2 dimensions are included, the network seems to be random since the degree distribution is characterized by a Poisson distribution. Data in Table 1 reports which subsets of observables in the 8-dimensional space still gives a reasonable scale-free approximation, with an exponent lambda closest to the value of 2.3 and a rms better or equal to 0.980, extracted from the full data set and with a reasonable fit. For scale-free networks the slope λ is generally between 2.1 and 3 [23], [32]. Barabasi and Albert [23] have analyzed the distribution function of connectivities for various large networks: actor collaboration network, www, and the citation patterns of the scientific publications. The respective exponents found were: λ_actor_ = 2.3±0.1, λ_www_ = 2.1±0.1, and λ_cite_ = 3. For relatively modest sized networks like the electrical power grid of the western USA with only 4941 vertices, the scaling region is less prominent but is nevertheless approximated by a power law with an exponent λ_power_ = 4.

In Figure 3(C) is shown that for rank 1 cells (cells where only one amino acid pair has been found) there is neither distinct distance nor solvent exposure preferred by the amino acid pair. The pairs present in cells of rank 1 [Fig. 3(C)] reflect the statistical preferences for residues being buried. In contrast, in Figure 3(D) it is seen that the inter-residue distance of 3.8–4.3Å is dominating the observations for rank ≥50 as well as a very low solvent accessibility – this is consistent with hydrophobic contact between the two linked residues. A distance of 4–5Å is often used in protein structural analysis to indicate a structural contact. Data displayed in Figure 6 and Figure 7 confirms that the most abundant amino acid pairs are indeed formed between Ala, Ile, Leu and Val residues. Interestingly, the next most preferred residues are Phe and Gly [Fig. 5(B) and Fig. 7A]. Data in Figure 5 shows that despite the higher occurrence of these four residues in pairs of amino acid residues in proteins, these are not the four most abundant residues in proteins. The same applies to Phe. Furthermore, data displayed in Figure 5C (distribution observed in the randomized dataset) shows that the peaks displayed in Figure 5B are statistically relevant: the clear preference for Ala, Ile, Leu and Val in amino acid pairs is not observed in the randomized matrix [Fig. 5(C)] while it was clear in the original non-randomized 8D matrix [Fig. 5(B)]. In Figure 5 we want to highlight that the occurrence of single amino acids is distinct from the occurrence of pairs of amino acids containing a particular amino acid. Our data strongly indicates that these pairs should be given much more attention than is currently the case. Please notice that Ile is not among the 10 most abundant residues in proteins [Fig. 5(A)] but is the third most abundant amino acid involved in pairwise interactions with other residues [Fig. 5(B)]. In Figure 3B we show the distribution of the amino acid pair containing residues for cells with rank ≥50 (cells where the number of pairs is ≥50; a total of 1.07*10^6^ amino acid pairs are observed). Unlike Figure 5, we now demand the each cell in the matrix containing information about each pair of amino acids has at least 50 pair occurrences. And we can see that the message is now even more distinct from the distribution of the single amino acids observed in Figure 5A. Please see Figure 3B. There is no doubt that proteins favor the pairwise interactions between Ala, Ile, Leu, and Val and that this preference is not due to the natural occurrence of single amino acids in nature. Please see that message depicted in Figure 5A and Figure 3B. We see in panel 3D that those residues (Ala, Ile, Leu, and Val) are buried (SA ≤10%).

The rank 1 residue pairs constitute a set of pairs for which a unique environment was found. Curiously, this set encompasses the vast majority of all residues on the protein surface, thus any amino acid pair on the protein surface is likely to represent a unique structural environment. Most sequence alignment methodologies will disregard sequence matches between the four most connected residues (Ala, Ile, Leu and Val). The argument has been that such residues are very abundant in the protein core and therefore provide little guidance for the alignment process. The data presented in the present paper suggests that whereas the single Ala, Ile, Leu or Val may be of little relevance, closely spaced pairs of such residues in space is the single most abundant feature in the data cube. A 3D structural prediction methodology that incorporates both the 1D sequence information with secondary structure information and with the new insight into the importance of the highly connected residues should be investigated.

The ratio plot displayed in Figure 7B (ratio between the number of each type of pairs found in the protein dataset and the number of pairs found in the randomized dataset) shows the statistical relevant of each type of pair. Data confirms that the most abundant pairs observed displayed in Figure 6 and Figure 7 are also statistically significant.

In Figure 8 is shown that the secondary structure of the amino acid pairs also vary with rank in a scale free manner. At rank = 1 all 4 types of secondary structures are well populated. At rank ≥50 (red vertical line) no amino acid pairs are found in the turn category, and coil is significantly less populated than both alpha and beta categories. From the analysis of the amino acid distribution [Fig. 3 (A,B)] we know that the four residues Ala, Ile, Leu and Val dominate the cells with rank ≥50, and that they occur largely buried (SA≤10%) in hydrophobic contact (3.8–4.3Å) (Fig 3D). We conclude that the four residues predominately located in alpha helices or beta strands form hydrophobic clusters, which provide the structural core of the protein structure. We also know that the amino acid pairs are separated by more than 4 residues. We conclude that these pairs are involved in through space contacts between different alpha-helices, beta-strands or a combination thereof. Data displayed in Figure 8 confirms that pairs containing the residues with the highest number of links (Ile, Leu, Val and Ala) connect different secondary structural elements (alpha-helices and beta-sheets), contributing in a significant way to protein structural stability.

Jha et al. [33] have reported a knowledge-based approach for determining the effective interactions between amino acids based on amino acid type, their secondary structure and the contact based environment that they find themselves in the native structure as measured by the number of neighbors. One major difference between this work and our present work is that they compute the number of connections based on the Cα-Cα connections while in our study we report contacts made through the functional groups of each amino acid side chain. This is an important difference. However, they find that the probability of contact of amino acids from the same type of secondary structures is higher in the case of helix and sheet, whereas for residues in the loop structure, the interacting residues are distributed in all types of secondary structures. Amino acid residues L, A, E, V, L, R and K make more contacts within helices and V, L, I, A, T, and F dominate in beta-sheets. G, P, A, S, and D amino acids favor contacts within loops. In our study, we also see that the most frequent interactions among residues happen among hub residues (L, I, V, A) when they are located in helices and beta-sheets. These very frequent interactions do not happen when they are located in turns or loops. Both studies find that interactions critical to keep protein fold involve the presence of hub residues (L, I, V, A) in helices and beta-sheets.

### Protein Structure Network: Small Scale-network or Scale-free Network

We will now further correlate our data with the previously published data and discuss the nature of protein networks. The dataset used by Brinda and Vishveshwara [34] in their analysis consisted of 232 globular proteins structures obtained from PDB. This dataset was non-redundant with sequence identity <20%. Each protein is represented as a graph consisting of a set of nodes and edges. Each amino acid in the protein structure is represented as a node, and the nodes (amino acids) are connected by edges based on the strength of non-covalent interactions between the side chains of the two amino acid residues. The strength of interaction between two amino acid side chains is evaluated taking into account the number of distinct atom pairs between the side chains of two interacting amino acid residues which come within a distance of 4.5Å. A hub is defined as a residue that established more than 4 contacts. Their analyses of the distribution of the nodes with *k* links as a function of the interactions criterion shows that above a certain cutoff, the plots show a power law tail with the critical exponent λ ranging from 1.2–2.3. Below that critical value the protein network seems to be random. When they investigate the preferences of different type of amino acids they observe that charge-delocalized planar side chains of Phe, Tyr, Trp, Arg, and His along with Met are preferred as strong hubs at higher interactions cutoffs, whereas the hydrophobic side chains of Leu, Ile, and Val, preferred as weak hubs, appear only at lower interaction cutoff. They do not state with which amino acid residues the hubs pair with and do not show the frequency of such contacts. They report that most hubs belong to the regular secondary structural regions of helices and sheets though the loops, turns, and the unassigned regions are not excluded at any interaction cutoff. In our study, we see that the most frequent interactions among hub residues (Val, Ala, Ile, Leu) happen when they are located in helices and beta-sheets (see Figure 8B). Less frequent interactions can happen when they are located in coils and they are not observed in turns. In our study we use a set of 8272 non-redundant pdb entries vs 232 used in the study by Brinda and Vishveshwara [34]. The methodology used in our study is different. We characterize an interacting pair of amino acid residues in terms of the previously mentioned 8 dimensions. The number of pairs found in a particular environment is stored in a matrix cell in an 8D data cube. When plotting the cell population against the number of cells that have the same population size, a scale free organization is found: ω(R) = R^−λ^, where R is the rank or population of a cell, and ω(R) is the number of times such a cell population was encountered [19]. When plotting log_2_(ω(R)) against log_2_(R), a straight line with slope −2.3 is obtained [Fig. 1(A)]. When analyzing which amino acid paired residues contributed to the cells with a population above 50, pairs of Ala, Ile, Leu and Val dominate the results. This result is statistically highly significant. We postulate that such pairs form “structural stability points” in the protein structure. Those are the so called hub residues. It is important to highlight that the way different authors arrived at the definition of “hub” might be different. Both our work and the work from Brinda and Vishveshwara^34^ leads to the conclusion that the hydrophobic side chains of Leu, Ile, and Val are hub residues. In our work we additionally show with which amino acid residues the hubs pair with and show the frequency of such contacts. Furthermore we show that the network of pairwise interactions has a scale-free nature. Our data shows that pairs of Ala, Ile, Leu and Val dominate the results are in buried α-helices or β-strands, in a spatial distance of 3.8–4.3Å and in a sequence distance >4 residues (please see Figure 6 and Figure 8 B). The location of the hubs can sometimes include coils, in the cases of less frequent pairs (blue areas in Figure 8B). Brinda and Vishveshwara [34] report that most hubs belong to the regular secondary structural regions of helices and sheets though the loops, turns, and the unassigned regions are not excluded at any interaction cutoff. We speculate that the scale free organization of the 8D protein fold structure combined with the clear dominance of Ala, Ile, Leu and Val is important for understanding the very nature of the protein structure formation. Our observations suggest that protein structures should be considered as a higher dimensional organization.

The work by Bagler and Sinha [35] reports that proteins show small-world network property, regardless of their structural class. This is based on the definition of “small world network”, since their L(average shortest path length)-C(average clustering coefficient) plots show a high C value and L scales logarithmically with N (number of nodes). In their paper the nodes were the Cα atoms of each residue. However, the degree distributions reported for the different classes of protein folds are characterized by a Poisson distribution, which points at random networks and not scale-free networks. It has been suggested that one of the main reasons for deviations from a scale-free connectivity distribution is the limited capacity of a given node [36]. A very important point is that in Bagler and Sinha [35] study they have used the Cα atoms of the amino acid as a node and two such nodes are said to be linked if they are less than or equal to 7Å. This analysis ought to give very different results if the nodes are defined as an atom belonging to the side chain. It is the different side chains that make the amino acid residues different and interactions among side chains are crucial for defining and keeping the protein fold, and any information on their connections is lost in an analysis based solely on the Cα atoms. Our observations are also shared by Greene and Higman [32]. The bulk of the interactions made by one residue are made through its side chain. Both in our work and in Brinda and Vishveshwarás work [34] we have considered the interactions between the side chains of the two amino acid residues, and we both see that the distribution of the “number of nodes with k links” as a function of the “number of links” is a scale free distribution at high interaction cutoff. Interestingly, the works of Bagler and Sinha^35^, Atilgan et al. [37], and Vendruscolo et al. [38] report that proteins have small-work network properties and their analyses had considered Cα or Cβ atoms as nodes, instead of atoms in the residueś side chains. There seems to be correlation between the nature and degree of connectivity of the node (if the node is less connected atom such as Cα, Cβ or a more connected atom belonging to the side chain of the amino acid residue) with the observed nature of the degree distribution: if small scale-network or scale-free network. If the nodes are less connected, like in the case of Cα or Cβ atoms, the study reveals that proteins are small-scale networks. On the other hand, if the nodes establish a larger number of connections, such as when the nodes include the side chains of the amino acid residues, than the scale-free nature of proteins is revealed. This observation is supported by the work by Amaral et al. [36] which report that one of the possible reasons for such a rich range of possible structures for small-world networks (scale-free networks are also small-world networks) is the capacity of a node to establish connections.

### The Functional Importance of “High Degree Nodes”

Very diverse organizations in nature and society such as social networks [39], scientific collaboration networks [40], metabolic networks [17] and human mobility [41] have all been found to exhibit scale free behavior. The World Wide Web is a scale free structure with hubs and nodes, where there are a few hubs with many millions of links and many nodes with few links. This type of structure has been shown to be very robust towards random errors and attacks [16]. It has been a common practice in the analyses of protein interaction networks to define an *ad hoc* threshold or degree scale such that all nodes (proteins) that have degree higher than this threshold are considered to be special in some sense and are called “hub” nodes. The notion of a hub protein is a special one because hub proteins, though defined arbitrarily, often do have special biological properties: they tend to be more essential than non-hub proteins [42], [43]. In spite of the scale-free degree distribution that characterizes most protein interaction networks, it is common to define an *ad hoc* degree scale that defines “hub” proteins having special topological and functional significance. This raises the concern that some conclusions on the functional significance of proteins based on network properties may not be robust [44]. The rules for identifying hubs in protein interaction networks are still being discussed. Just as the sharp rise in connectivity at a certain degree defines a degree “scale” that can be used to differentiate hubs from non-hubs, other centrality measures could have characteristic scales in protein interaction networks, such has concepts that include the functional significance of the protein. In our paper we analysed the interaction network in a protein, i.e., the residue-residue interaction network and we showed that it also has a “scale-free” property, since the distribution of the amino acid pairwise interaction is a power-law distribution. Indeed some amino acid residues pair or interact much more frequently that others and the frequency of those interactions is observed to be scale-free. Furthermore, we correlated the observation that 4 amino acid residues (the 4 high-degree “nodes”) display significantly larger number of contacts with other amino acids with the biological significance of this observation, namely the important of such interaction among super-hydrophobic and hydrophobic residues for protein structural stability. In our study, the 4 amino acid residues (high degree nodes) with the high number of through space contacts done are known to be superhydrophobic (Ile, Leu and Val) and hydrophobic (Ala) residues and the many interactions carried out by those few residues do play an important role in protein structural stability. It is not surprising to find a high proportion of hydrophobic amino acids in the protein core – but it is surprising that the packing of the four most abundant paired residues is restricted to relatively few (Ala, Ile, Leu and Val). So, the functionality or purpose of the above mentioned “highly linked nodes” is clear in a protein inner network. Interestingly, out of the 4 “high-degree” residues, it is the 3 super hydrophobic ones that establish the larger number of interactions with other amino acids. Leu has the high-degree connectivity followed by Val, Ile and at last Ala (please see 2D insert in Figure 6). We can also see that all four residues have the larger number of interactions with Leu, which is interesting.

An important question for the community that studies protein-protein interaction networks is what leads to the high connectivity of hub proteins. Ekman et al report that there is an enrichment of multi-domain proteins among the hub proteins compared to non-hub proteins, and they are, on average, longer [26]. Moreover, repeated domains are clearly overrepresented in hub proteins. The presence of repeated domains and multiple domains in hubs may partly explain their high connectivities. It is evident that domain repeats, which are associated with binding, are enriched in hubs. The evolutionary origin of scale-free networks is probably rooted in gene duplication [45]–[50]. In a parallel way, the reasons for the large connectivities seen among the reported 4 residues (Ala, Ile, Leu, Val) can be discussed. Among those 4 residues, the 3 amino acid residues that display the largest number of pairwise interaction are Ile, Leu and Val. These residues are known to be very hydrophobic amino acids. Ala is also hydrophobic. Interestingly, none of these residues have a functional side chain critical, for example, for catalysis. These residues through the establishment of a large number of hydrophobic interactions mediated by their side chains contribute significantly to protein structural stability. The large number of such interactions contributes to protein stability. It can be observed from Figure 3D that those residues that display many pairwise interactions are located in buried regions of the protein (solvent accessibilities between 0 and 10%), indicating that their contribution to protein stability is achieved at the proteińs core. Furthermore, they are preferentially located in αhelices and β-sheets, in a sequence distance >4 residues, at a preferred distance of 3.8–4.3Å, which is consistent with hydrophobic contacts between the interacting residues. This is important in order to achieve the proteińs 3D structure and secure structural stability.

### Conclusion

The present work has demonstrated that scale free organization characterizes amino acid pair interactions in proteins. Several other authors have addressed scale free aspects of protein structures [51]–[54] but at a higher level of complexity such as structural diversity, fold identification or protein functionality. This is the first report in literature documenting that amino acid pair interactions in proteins are organized in a scale free manner. We suggest that 3D structure prediction methodologies should also incorporate the new insight into the importance of the highly connected residues presented in this paper.

## Methods

### Protein Dataset

A list of high resolution protein chains (resolution ≤3.0 Å) with sequence identity ≤35% was retrieved from the Pisces server [19]. All structures had a minimum chain length of 40 and a maximum R value – a measure of how well the experimental data can be predicted from the refined model - of 1.00. Non-X-ray structures and structures only with Cα atoms were excluded. The Pisces culling method selected was “chain”. The downloaded list contained 9039 chains, present in 8598 different.ent files. The.ent files were downloaded from the Research Collaboratory for Structural Bioinformatics (RCSB) [20]. The corresponding.hssp files were downloaded from the homology-derived secondary structure of proteins (HSSP) database [21]. Entries in the Pisces list for which the corresponding.hssp files were not available were discarded, leaving 8272.ent files with corresponding.hssp files. These files contained 8706 of the non-redundant chains from the Pisces list.

In our analysis we only included experimental PDB structures. We have not included homology derived structures in order to achieve a larger dataset. The.hssp file associated to each protein structure file (.ent file) has been downloaded simply in order to know in which secondary structural element (alpha helix, beta strand, coil or turn) each amino acid was located. This information was needed in order to display the data presented in Figure 8.

### Software

One software package called ProExtract was developed [22]. ProExtract combined the data from.ent and.hssp files into MATLAB structures, which were saved in.mat format (a MATLAB data file). For each.ent file, the atoms’ coordinates and chain information were loaded into ProExtract, while information on residue type, secondary structure and solvent accessibility (SA) was loaded from the corresponding.hssp file. Since many.ent and.hssp files were found to contain errors, ProExtract included a validation routine, where residues as a minimum were required to have information on the Cα and functional atoms coordinates (*vide infra*), residue type, secondary structure, solvent accessibility (SA) and chain length. Furthermore,.hssp entries were required to have information about which residue and chain they corresponded to in the.ent file, as numbering in.hssp and.ent files might differ. Residues that did not have all the required information were discarded, while the rest of the chain information was retained. Those that were accepted were added to the MATLAB structure file for that protein. As a result, a file for each protein was created containing combined information on atom coordinates and chains, residue types, secondary structure and SA.

The program ProExtract has been made accessible. ProExtract was developed using MATLAB v7 (2010a). The source code of the program ProExtract (used to create the 8D tensor) has been uploaded as supplementary information. The file names are: “Information S1” and “Figure S1”. The description on how to run the software ProExtract can be found in the file “Instructions S1”. In order to run ProExtract two input files are needed: the protein.ent list and the list of correspondent hssp files. A file named “Information S2” has been uploaded as supplementary information, where the name of all pdb files has been listed. This file should be open with WordPad. The associated.ent and.hssp files are publically available.

As output, ProExtract created a database in the form of an 8D tensor from the.mat files. The tensor contained information about pairs of amino acids present in the different SA protein shells. Two amino acid residues were considered a pair if they belonged to the same chain, were within the same SA-bin and had a distance less than 8.25Å between their functional atoms (*vide infra*). Each of the eight tensor directions was binned according to:

1. Type of the first amino acid (AA1) (20 bins)
2. Type of the second amino acid (AA2) (20 bins)
3. Solvent accessibility of the amino acid pair (SA) (12 bins)
4. Distance between atoms in functional groups (D) (14 bins)
5. Secondary structure for the first amino acid (SS1) (4 bins)
6. Secondary structure for the second amino acid (SS2) (4 bins)
7. Chain length (CL) (12 bins)
8. Sequence distance between AA1 and AA2 (SD) (6 bins)

Please notice that the first and second tensor directions are not the probability of occurrence of the amino acids in proteins. We are not addressing the occurrence of individual amino acids – we are addressing pairs of amino acids, enumerated only if the two component amino acids are found in a mutual distance of less than 8.25Å and if they are found in the same solvent accessibility bin. See bin definitions in section “*Bin definitions and functional atoms*”. 8272.mat files were processed successively. All possible combinations of two residues were carried out to test if the two residues would constitute a pair (*vide supra*). When a pair was identified, the count in the data tensor cell with the coordinates (AA1, AA2, SA, D, SS1, SS2, CL, SD) was increased by one. A total of 5.211.796 pairs were identified. These were distributed between 1.756.714 cells in the tensor.

As output, ProExtract created an index dataset which could be used to identify the specific interactions that gave rise to the counts in a tensor cell. The index set was an 8D MATLAB cell character array. Whenever a pair was registered, a string was added to the corresponding cell in the index array of the form “1ABC0102A1030B” for the imaginary pair of amino acids 102A and 1030B in Protein Data Bank (PDB) structure 1ABC. When more than one pair was registered in the same cell, a new line was created for each pair in the cell. In this way it was possible to retrieve the protein(s) as well as the local structural context around an amino acid pair that contributed to the count in a particular cell.

In order to determine the statistical significance of the coordinates of each of the pairs in a protein, ProExtract shuffled the amino acid residues maintaining the amino acid composition of each protein. This process was repeated 10 times for each of the 8272 proteins, and the resulting 8D tensors were averaged. The average 8D tensor was used as a reference dataset. For a given pair, the ratio between the actual count in a cell in the observed 8D tensor and the average count in the reference dataset was a measure of the significance of the cell. We have computed the ratio between the actual findings and the randomized value [Fig. 5(B,C)].

### Bin Definitions and Functional Atoms

The first dimension of the dataset tensor had 20 amino acid bins: Ala, Arg, Asn, Asp, Cys, Gln, Glu, Gly, His, Ile, Leu, Lys, Met, Phe, Pro, Ser, Thr, Trp, Tyr, Val. The second dimension had 20 amino acid bins, identical to the first dimension. The third dimension had 12 solvent accessibility bins (SA in %): SA≤0, 0<SA≤10, 10<SA≤20, 20<SA≤30, 30<SA≤40, 40<SA≤50, 50<SA≤60, 60<SA≤70, 70<SA≤80, 80<SA≤90, 90<SA≤100, SA>100. The fourth dimension had 14 distance bins (D in Å): D≤1.75, 1.75<D≤2.25, 2.25<D≤2.75, 2.75<D≤3.25, 3.25<D≤3.75, 3.75<D≤4.25, 4.25<D≤4.75, 4.75<D≤5.25, 5.25<D≤5.75, 5.75<D≤6.25, 6.25<D≤6.75, 6.75<D≤7.25, 7.25<D≤7.75, 7.75<D≤8.25. The fifth dimension had four secondary structure bins for AA1: α-helix, β-strand, turn and coil. The sixth dimension had four secondary structure bins for AA2, identical to the fifth dimension. The seventh dimension had 12 chain length bins: CL≤0, 0<CL≤100, 100<CL≤200, 200<CL≤300, 300<CL≤400, 400<CL≤500, 500<CL≤600, 600<CL≤700, 700<CL≤800, 800<CL≤900, 900<CL≤1000, CL>1000. The eighth dimension had 6 sequence distance bins: 0, 1, 2, 3, 4, >4.

The functional atoms were for Ala CB, Arg NH1 and NH2, Asn ND2 and OD1, Asp OD1 and OD2, Cys SG, Gln NE2 and OE1, Glu OE1 and OE2, Gly CA, His ND1, Ile CG1 and CG2, Leu CG, Lys NZ, Met SD, Phe CZ, Pro CG, Ser OG, Thr OG1, Trp CE2, Tyr OH, Val CG1 and CG2 (atom nomenclature as described in the.ent files).

## Supporting Information

Figure S1 **Source code associated file.** This is the user interface that is automatically displayed in MATLAB when we open and run the source code file “Information S1”.
(FIG)

Information S1 **Source code file.** The source code of the program ProExtract (.m file, version 2.4) is listed in the file “Information S1” with associated file “Figure S1”. These files can be open in MATLAB.
(M) Click here for additional data file.

Information S2 **List of** **PDB files.** The two input files needed in order to run ProExtract are the protein.ent list and the list of correspondent hssp files. A file named “Information S2” contains the name of all pdb files that have been used. This file should be open with WordPad. The associated.ent and.hssp files are publically available.
(TXT)

Instructions S1 **How to run the software ProExtract.** Detailed description of all files needed and how to run the program ProExtract.
(DOC)
