# Supplementary material for: Scale-Free Behaviour of Amino Acid Pair Interactions in Folded Proteins
Source: PLoS One. 2012 Jul 26;7(7):e41322. doi: 10.1371/journal.pone.0041322 (PMC3406053; doi:10.1371/journal.pone.0041322)

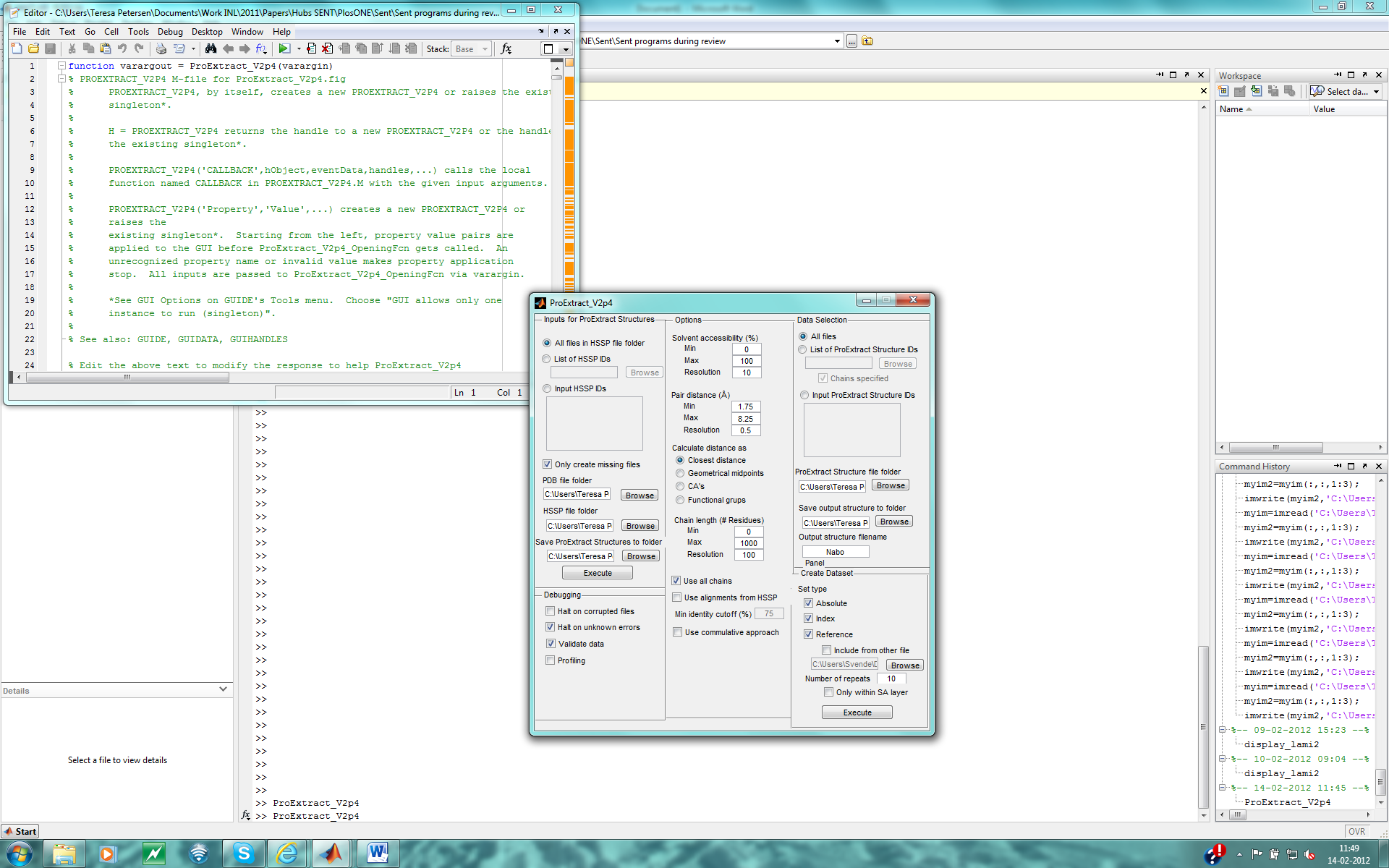


This is the **ProExtract_V2p4.fig -** user interface that is automatically opened when we open (in MATLAB) and run the source code file ProExtract_V2p4.m

After opening source code file ProExtract_V2p4.m, click here in order to Run it.

Manual for the ProExtract module

Written in MATLAB version 2009

Load the program ProExtract_v2p4.m into Matlab. Run the program. The file ProExtract_v2p4.fig should be located in the same directory as ProExtract_v2p4.m.

User instructions:

1. Download all HSSP and PDB files that are to be used for calculations. Save all HSSP files in one folder and all PDB files in another folder.
2. First of all, ProExtract structure files must be created (created (temporary structure files that are used to create the 8D dataset in the end). The upper left corner of the program will be used. Chose the option “All files in HSSP file folder”.


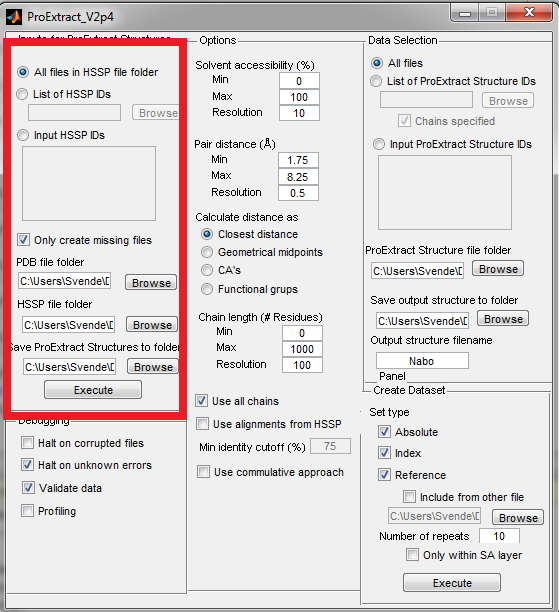


1. Point the “PDB file folder” to the folder where the PDB files are saved, likewise for the “HSSP file folder”. Make an empty folder for ProExtractStruct Files and point “Save ProExtract Structure to” to it.
2. Hit “Execute” (the upper left one) and wait till the calculations finishes.
3. Now the actual dataset can be calculated. Adjust the options in the middle column according to needs.


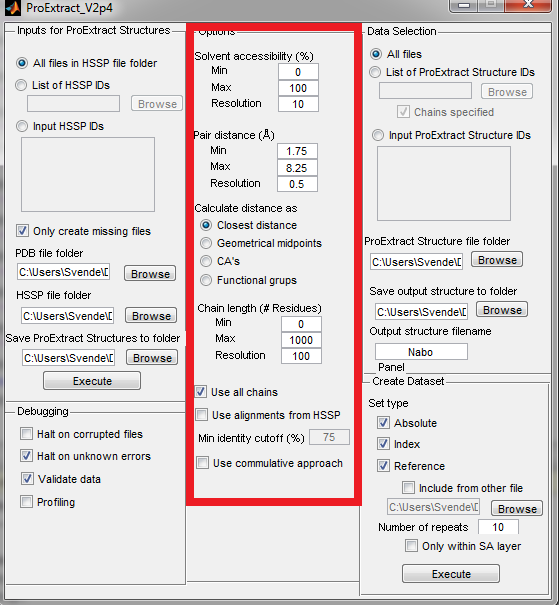


1. In the upper right corner, select “List of ProExtract Structure IDs” and point the program to the list of chains to be used from Pisces “hsspok_pisces_35_id.txt”. Point the program to the folder from point 3, where the ProExtractStruct Files were saved.


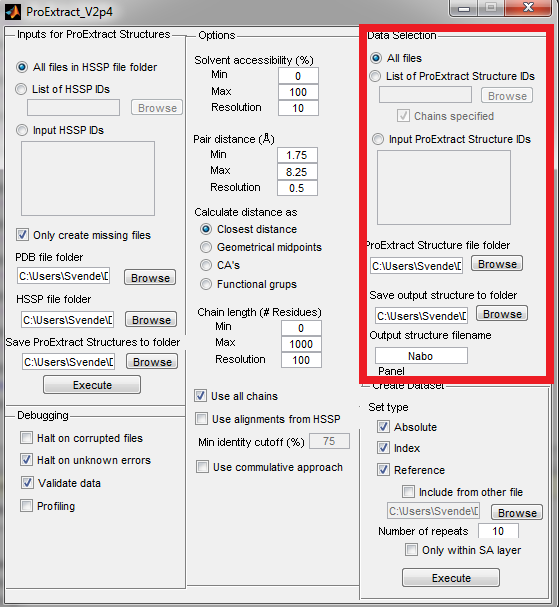


Output structure is the name of the file that contains the 8D datasets.

This file contains all 3 datasets. It will have the filename entered into “output structure”.

1. Select output folder and name of the dataset file to be created.
2. Press Execute in the bottom right corner.


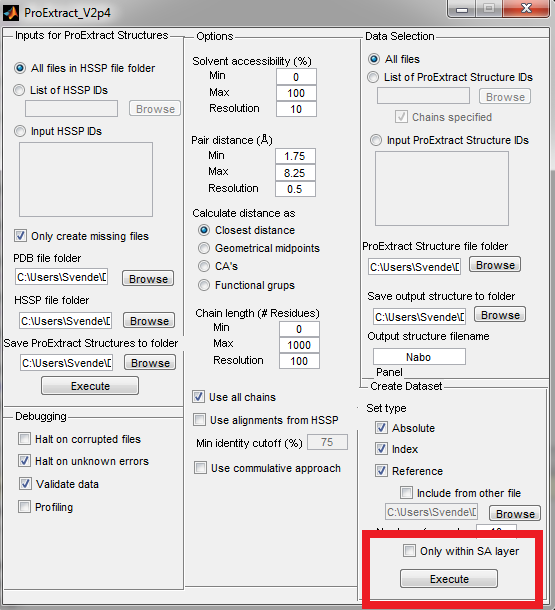

Supplement: Instructions S1 — How to run the software ProExtract. Detailed description of all files needed and how to run the program ProExtract. (DOC) [file pone.0041322.s004.doc]
